# Crystal structure of aqua­(nitrato-κ*O*)dioxido{2-[3-(pyridin-2-yl-κ*N*)-1*H*-1,2,4-triazol-5-yl-κ*N* ^4^]phenolato-κ*O*}uranium(VI) aceto­nitrile monosolvate monohydrate

**DOI:** 10.1107/S205698901502438X

**Published:** 2016-01-06

**Authors:** Oleksandr Vashchenko, Ilona Raspertova, Viktoriya Dyakonenko, Svitlana Shishkina, Dmytro Khomenko, Roman Doroschuk, Rostislav Lampeka

**Affiliations:** a Department of Chemistry, Taras Shevchenko National University of Kyiv, 64/13, Volodymyrska Str., Kyiv, 01601, Ukraine; b STC "Institute for Single Crystals", National Academy of Science of Ukraine, 60 Lenina Ave., Kharkiv 61001, Ukraine

**Keywords:** crystal structure, uran­yl(VI) ion, 1,2,4-triazole

## Abstract

In the title compound, [U(C_13_H_9_N_4_O)(NO_3_)O_2_(H_2_O)]·CH_3_CN·H_2_O, the U^VI^ atom is seven-coordinated in a distorted penta­gonal–bipyramidal N_2_O_5_ manner by one tridentate triazole ligand, one monodentate nitrate anion and one water mol­ecule in the equatorial plane and by two uran­yl(VI) O atoms in the axial positions. In the crystal, the U^VI^ complex mol­ecule is linked to the water and aceto­nitrile solvent mol­ecules through N—H⋯N, O—H⋯O and O—H⋯N hydrogen bonds, forming a sheet structure parallel to the *bc* plane. The sheets are further linked by an additional O—H⋯O hydrogen bond, forming a three-dimensional network.

## Abstract 2

The U^VI^ atom exhibits a penta­gonal–bipyramidal N_2_O_5_ coordination environment. In the complex, the 1,2,4-triazole ligand is coordinated in a tridentate manner.

## Chemical context   

The synthesis of coordination compounds with *N*-donor heterocyclic ligands is one of the fastest growing areas of coordination chemistry. 1,2,4-Triazoles and their derivatives can be assigned for such types of ligands. The presence of the 1,2,4-triazole ring in the organic ligand provides an additional site for coordination (Aromí *et al.*, 2011 ▸). The presence of additional donor groups in the 3- and 5-positions of the triazole moiety provides a greater number of possibilities for chelation of metal ions, involving tridentate bis-chelate functions.
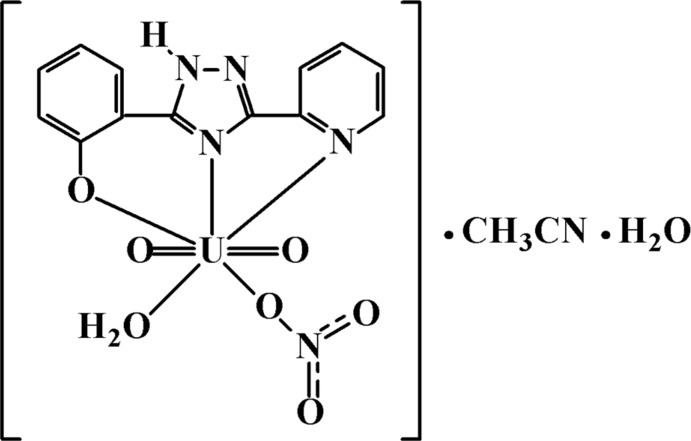


It should be noted that UO_2_^2+^ complexes with such types of ligands have rarely been investigated. Thus, only three uranyl complexes with 1,2,4-triazole derivatives have been characterized (Daro *et al.*, 2001 ▸; Weng *et al.*, 2012 ▸; Raspertova *et al.*, 2012 ▸). As part of our continuing study of uranium coordination compounds with nitro­gen-donor ligands (Raspertova *et al.*, 2012 ▸), we report here the structure of the title compound.

## Structural commentary   

**Figure 1:**
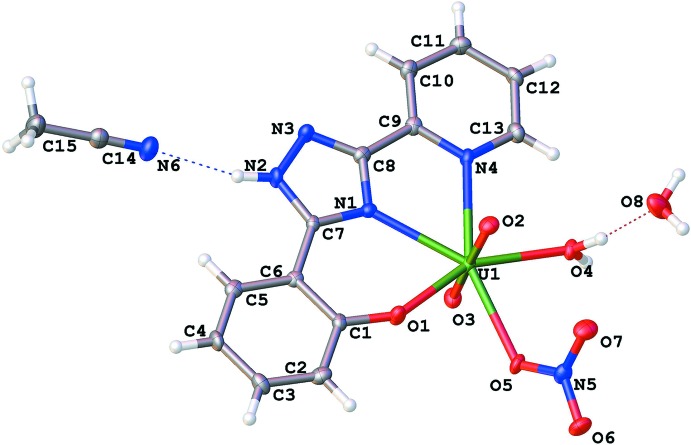
The mol­ecular structure of the title compound, shown with 50% probability displacement ellipsoids. Dashed lines indicate hydrogen bonds.

**Figure 2:**
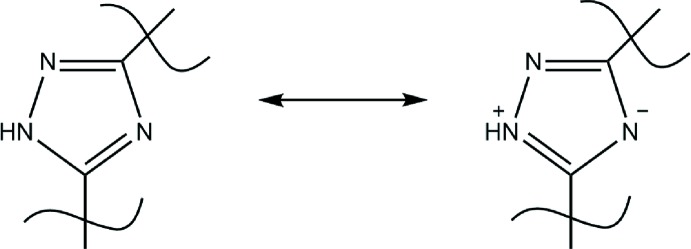
Scheme showing two possible resonance structures in the triazole ligand.

The coordination polyhedron of the U^VI^ atom in the title complex is a distorted penta­gonal bipyramid. It is coordinated in a tridentate manner by the 1,2,4,-triazole ligand together with the water mol­ecule and the monodentate nitrate anion in the equatorial plane. Two oxido ligands are placed in the axial positions (Fig. 1 ▸). The U1—O1 bond length [2.206 (3) Å] is comparable with those reported for related six-membered chelate fragments involving phenolate and *N*-atom donors (Sopo *et al.*, 2008 ▸; Ahmadi *et al.*, 2012 ▸). The U—N bond lengths [2.489 (4) and 2.658 (4) Å] are consistent with the situation in other pyridine-bonded uranium complexes (Amoroso *et al.*, 1996 ▸; Gatto *et al.*, 2004 ▸). The uranyl group is not exactly linear [O2=U1=O3 = 175.36 (14)°]. Non-linear O=U= groups are generally found in uranyl complexes with five non-symmetrically bonding equatorial ligands. All non-hydrogen atoms of the organic ligand are coplanar within 0.01 Å. The N1—C7 and C7—N2 bond lengths of the triazole ring are equalized [1.336 (5) Å for both]. This value is longer than a C*sp* ^2^ =N double bond (1.276 Å) and shorter than a C*sp* ^2^—N single bond (1.347 Å) (Orpen *et al.*, 1994 ▸). It can be assumed that the structure of the triazole ring is the superposition of two possible resonance structures as shown in Fig. 2 ▸.

## Supra­molecular features   

**Figure 3:**
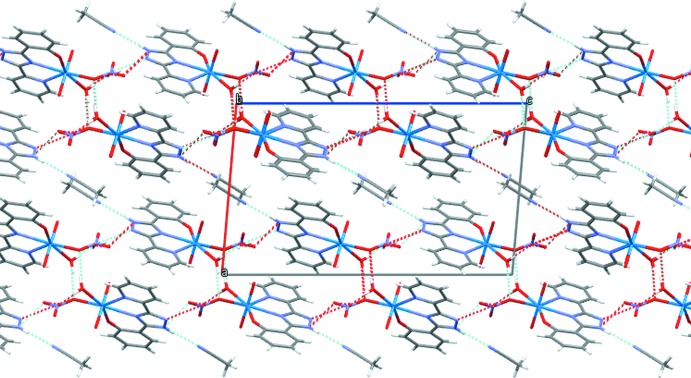
Packing diagram of the title compound, viewed along the *b* axis. Inter­molecular hydrogen bonds are shown as dashed lines.

In the crystal, the complex mol­ecule is linked to the water and aceto­nitrile solvent mol­ecules through N2—H2⋯N6, O4—H4*B*⋯O8, O8—H8*A*⋯N3^ii^ and O8—H8*B*⋯O6^iii^ hydrogen bonds (symmetry codes in Table 1 ▸), forming a sheet structure parallel to the *bc* plane. The sheets are further linked by an O4—H4*A*⋯O5^i^ hydrogen bond (Table 1 ▸), forming a three-dimensional network (Fig. 3 ▸).

## Database survey   

In the Cambridge Structural Database (Version 5.36, November 2014; Groom & Allen, 2014 ▸), only three uranyl complexes with derivatives of 1,2,4-triazole are reported (Daro *et al.*, 2001 ▸; Weng *et al.*, 2012 ▸; Raspertova *et al.*, 2012 ▸). 72 structures containing a 5-pyridin-1*H*-1,2,4-triazole fragment are found. A search for the 3-hy­droxy­phenyl-1,2,4-triazole fragment yielded 14 hits, including: 2,2′-[1-(2,4,6-tri­chloro­phen­yl)-1*H*-1,2,4-triazole-3,5-di­yl]diphenol (Li *et al.*, 2008 ▸); 2-[5-(2-pyrid­yl)-1,2,4-triazole-3-yl]phenol 2-[3-(2-pyrid­yl)-1,2,4-triazole-5-yl]phenol bis­(7,7,8,8-tetra­cyano­quinodi­methane) (Bentiss *et al.*, 2002 ▸); bis­[μ_2_-1-phenyl-3,5-bis­(2-oxyphen­yl)-1,2,4-triazole]bis­(pyridine)­dicopper (Steinhauser *et al.*, 2004 ▸). Only one compound containing both hy­droxy­phenyl and pyridyl, as substituents in the 3- and 5-positions of 1,2,4-triazole, was found (Bentiss *et al.*, 2002 ▸).

## Synthesis and crystallization   

A mixture of 3-(2-hy­droxy­phen­yl)-5-(pyridin-2-yl)-1*H*-1,2,4-triazole (0.5 mmol) and [UO_2_(NO_3_)_2_]·2H_2_O (0.5 mmol) in aceto­nitrile (20 ml) was stirred for 20 min. The solution was left to evaporate slowly at room temperature. Red single crystals suitable for X-ray analysis were obtained after 2 d.

## Refinement   

Crystal data, data collection and structure refinement details are summarized in Table 2 ▸. All hydrogen atoms were located in a difference Fourier map. The positional parameters of water H atoms were refined, with the restraint O—H = 0.860 (2) Å and the constraint *U*_iso_(H) = 1.5*U*_eq_(O). All other H atoms were constrained to ride on their parent atoms, with *U*_iso_(H) = 1.2*U*_eq_(C, N).

## Supplementary Material

Crystal structure: contains datablock(s) Global, I. DOI: 10.1107/S205698901502438X/is5435sup1.cif

Structure factors: contains datablock(s) I. DOI: 10.1107/S205698901502438X/is5435Isup2.hkl

CCDC reference: 1443165

Additional supporting information:  crystallographic information; 3D view; checkCIF report

## Figures and Tables

**Table 1:** Hydrogen-bond geometry (Å, °)

*D*—H⋯*A*	*D*—H	H⋯*A*	*D*⋯*A*	*D*—H⋯*A*
O4—H4*A*⋯O5^i^	0.86 (1)	1.92 (2)	2.752 (4)	162 (5)
O4—H4*B*⋯O8	0.86 (1)	1.73 (1)	2.581 (5)	168 (5)
N2—H2⋯N6	0.86	2.07	2.909 (5)	165
O8—H8*A*⋯N3^ii^	0.86 (1)	2.05 (2)	2.890 (5)	164 (6)
O8—H8*B*⋯O6^iii^	0.86 (1)	2.22 (3)	3.001 (5)	150 (6)

Symmetry codes: (i) 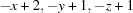; (ii) 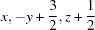; (iii) 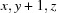.

**Table 2:** Experimental details

Crystal data	
Chemical formula	[U(C_13_H_9_N_4_O)(NO_3_)O_2_(H_2_O)]·CH_3_CN·H_2_O
*M*_r_	646.37
Crystal system, space group	Monoclinic, *P*2_1_/*c*
Temperature (K)	294
*a*, *b*, *c* (Å)	12.0962 (3), 7.87839 (17), 20.4041 (4)
β (°)	94.829 (2)
*V* (Å^3^)	1937.57 (7)
*Z*	4
Radiation type	Mo *K*α
μ (mm^−1^)	8.44
Crystal size (mm)	0.5 × 0.3 × 0.2

Data collection	
Diffractometer	Agilent Xcalibur, Sapphire3
Absorption correction	Multi-scan (*CrysAlis PRO*; Agilent, 2014 ▸)
*T*_min_, *T*_max_	0.055, 0.185
No. of measured, independent and observed [*I* > 2σ(*I*)] reflections	9385, 4446, 3936
*R*_int_	0.032
(sin θ/λ)_max_ (Å^−1^)	0.650

Refinement	
*R*[*F* ^2^ > 2σ(*F* ^2^)], *wR*(*F* ^2^), *S*	0.029, 0.064, 1.05
No. of reflections	4446
No. of parameters	284
No. of restraints	4
H-atom treatment	H atoms treated by a mixture of independent and constrained refinement
Δρ_max_, Δρ_min_ (e Å^−3^)	1.81, −0.88

Computer programs: *CrysAlis PRO* (Agilent, 2014 ▸), *SHELXT* (Sheldrick, 2015*a* ▸), *SHELXL2014* (Sheldrick, 2015*b* ▸) and *OLEX2* (Dolomanov *et al.*, 2009 ▸).

## supplementary crystallographic information

### Crystal data

**Table:**

[U(C_13_H_9_N_4_O)(NO_3_)O_2_(H_2_O)]·CH_3_CN·H_2_O	*F*(000) = 1216
*M**_r_* = 646.37	*D*_x_ = 2.216 Mg m^−^^3^
Monoclinic, *P*2_1_/*c*	Mo *K*α radiation, λ = 0.71073 Å
*a* = 12.0962 (3) Å	Cell parameters from 4414 reflections
*b* = 7.87839 (17) Å	θ = 3.3–31.9°
*c* = 20.4041 (4) Å	µ = 8.44 mm^−^^1^
β = 94.829 (2)°	*T* = 294 K
*V* = 1937.57 (7) Å^3^	Block, red
*Z* = 4	0.5 × 0.3 × 0.2 mm

### Data collection

**Table:**

Agilent Xcalibur, Sapphire3 diffractometer	4446 independent reflections
Radiation source: Enhance (Mo) X-ray Source	3936 reflections with *I* > 2σ(*I*)
Graphite monochromator	*R*_int_ = 0.032
Detector resolution: 16.1827 pixels mm^-1^	θ_max_ = 27.5°, θ_min_ = 3.1°
ω scans	*h* = −15→15
Absorption correction: multi-scan (*CrysAlis PRO*; Agilent, 2014)	*k* = −5→10
*T*_min_ = 0.055, *T*_max_ = 0.185	*l* = −26→26
9385 measured reflections	

### Refinement

**Table:**

Refinement on *F*^2^	4 restraints
Least-squares matrix: full	Hydrogen site location: mixed
*R*[*F*^2^ > 2σ(*F*^2^)] = 0.029	H atoms treated by a mixture of independent and constrained refinement
*wR*(*F*^2^) = 0.064	*w* = 1/[σ^2^(*F*_o_^2^) + (0.0276*P*)^2^] where *P* = (*F*_o_^2^ + 2*F*_c_^2^)/3
*S* = 1.05	(Δ/σ)_max_ = 0.001
4446 reflections	Δρ_max_ = 1.81 e Å^−^^3^
284 parameters	Δρ_min_ = −0.88 e Å^−^^3^

### Special details

**Table:**

Geometry. All e.s.d.'s (except the e.s.d. in the dihedral angle between two l.s. planes) are estimated using the full covariance matrix. The cell e.s.d.'s are taken into account individually in the estimation of e.s.d.'s in distances, angles and torsion angles; correlations between e.s.d.'s in cell parameters are only used when they are defined by crystal symmetry. An approximate (isotropic) treatment of cell e.s.d.'s is used for estimating e.s.d.'s involving l.s. planes.

### Fractional atomic coordinates and isotropic or equivalent isotropic displacement parameters (Å^2^)

**Table:**

	*x*	*y*	*z*	*U*_iso_*/*U*_eq_	
U1	0.81873 (2)	0.51832 (2)	0.40174 (2)	0.01286 (6)	
O1	0.7272 (3)	0.2815 (4)	0.37710 (15)	0.0193 (7)	
O2	0.6934 (2)	0.6205 (4)	0.41869 (15)	0.0189 (7)	
O3	0.9494 (2)	0.4323 (4)	0.38551 (15)	0.0179 (6)	
O4	0.9187 (2)	0.7071 (4)	0.47688 (15)	0.0164 (6)	
H4A	0.9886 (8)	0.709 (7)	0.489 (2)	0.025*	
H4B	0.890 (3)	0.768 (5)	0.5061 (16)	0.025*	
O5	0.8563 (2)	0.3461 (4)	0.50215 (14)	0.0186 (7)	
O6	0.8368 (3)	0.2773 (4)	0.60322 (15)	0.0234 (7)	
O7	0.7619 (3)	0.5091 (4)	0.56334 (18)	0.0258 (8)	
N1	0.7589 (3)	0.5025 (4)	0.28218 (18)	0.0128 (7)	
N2	0.6933 (3)	0.4457 (5)	0.18299 (18)	0.0162 (7)	
H2	0.6608	0.3920	0.1501	0.019*	
N3	0.7399 (3)	0.6016 (4)	0.17900 (18)	0.0159 (8)	
N4	0.8707 (3)	0.7819 (4)	0.32904 (18)	0.0134 (7)	
N5	0.8162 (3)	0.3796 (5)	0.55859 (18)	0.0174 (8)	
N6	0.5829 (3)	0.3255 (5)	0.0595 (2)	0.0266 (9)	
C1	0.6794 (3)	0.1782 (5)	0.3324 (2)	0.0138 (8)	
C2	0.6414 (3)	0.0177 (5)	0.3517 (2)	0.0184 (9)	
H2A	0.6503	−0.0141	0.3957	0.022*	
C3	0.5915 (3)	−0.0919 (5)	0.3057 (2)	0.0183 (9)	
H3	0.5661	−0.1965	0.3194	0.022*	
C4	0.5781 (3)	−0.0498 (5)	0.2396 (2)	0.0166 (9)	
H4	0.5443	−0.1250	0.2090	0.020*	
C5	0.6158 (3)	0.1056 (5)	0.2200 (2)	0.0155 (9)	
H5	0.6081	0.1343	0.1756	0.019*	
C6	0.6654 (3)	0.2206 (5)	0.2653 (2)	0.0126 (8)	
C7	0.7042 (3)	0.3861 (5)	0.2445 (2)	0.0114 (8)	
C8	0.7785 (3)	0.6308 (5)	0.2401 (2)	0.0142 (9)	
C9	0.8385 (3)	0.7813 (5)	0.2635 (2)	0.0142 (9)	
C10	0.8617 (3)	0.9142 (5)	0.2225 (2)	0.0163 (9)	
H10	0.8386	0.9105	0.1778	0.020*	
C11	0.9198 (3)	1.0527 (6)	0.2489 (2)	0.0184 (9)	
H11	0.9361	1.1435	0.2222	0.022*	
C12	0.9532 (3)	1.0555 (6)	0.3147 (2)	0.0169 (9)	
H12	0.9923	1.1478	0.3331	0.020*	
C13	0.9277 (3)	0.9180 (5)	0.3534 (2)	0.0176 (9)	
H13	0.9510	0.9201	0.3980	0.021*	
C14	0.5369 (4)	0.2758 (6)	0.0120 (2)	0.0199 (10)	
C15	0.4767 (4)	0.2097 (6)	−0.0472 (2)	0.0271 (11)	
H15A	0.4567	0.3017	−0.0767	0.041*	
H15B	0.4107	0.1531	−0.0358	0.041*	
H15C	0.5227	0.1308	−0.0682	0.041*	
O8	0.8271 (3)	0.9203 (5)	0.55170 (19)	0.0344 (9)	
H8A	0.811 (5)	0.899 (8)	0.5911 (11)	0.052*	
H8B	0.844 (5)	1.026 (2)	0.554 (3)	0.052*	

### Atomic displacement parameters (Å^2^)

**Table:**

	*U*^11^	*U*^22^	*U*^33^	*U*^12^	*U*^13^	*U*^23^
U1	0.01190 (9)	0.01556 (9)	0.01102 (9)	0.00016 (6)	0.00039 (6)	−0.00106 (6)
O1	0.0240 (17)	0.0209 (15)	0.0128 (16)	−0.0051 (14)	0.0006 (13)	0.0014 (13)
O2	0.0149 (15)	0.0223 (16)	0.0193 (16)	0.0039 (13)	0.0005 (13)	0.0015 (14)
O3	0.0129 (14)	0.0216 (15)	0.0190 (17)	0.0029 (13)	0.0000 (13)	−0.0040 (14)
O4	0.0138 (15)	0.0233 (15)	0.0120 (16)	0.0025 (14)	−0.0002 (13)	−0.0054 (13)
O5	0.0216 (16)	0.0249 (16)	0.0089 (15)	0.0012 (13)	−0.0011 (13)	−0.0012 (13)
O6	0.0261 (18)	0.0266 (17)	0.0173 (17)	0.0002 (15)	0.0008 (14)	0.0083 (15)
O7	0.0230 (17)	0.0289 (18)	0.0260 (19)	0.0110 (14)	0.0042 (15)	0.0026 (15)
N1	0.0115 (16)	0.0133 (17)	0.0135 (18)	0.0012 (14)	0.0007 (14)	0.0015 (14)
N2	0.0154 (17)	0.0184 (17)	0.0144 (19)	−0.0051 (15)	−0.0009 (15)	−0.0043 (16)
N3	0.0167 (18)	0.0177 (18)	0.0134 (19)	−0.0033 (15)	0.0017 (15)	0.0001 (15)
N4	0.0104 (16)	0.0174 (17)	0.0120 (18)	0.0003 (14)	−0.0015 (14)	−0.0026 (15)
N5	0.0140 (18)	0.0236 (19)	0.0150 (19)	−0.0034 (16)	0.0033 (15)	0.0007 (16)
N6	0.027 (2)	0.033 (2)	0.019 (2)	−0.0034 (19)	−0.0013 (18)	−0.0018 (19)
C1	0.0110 (19)	0.0156 (19)	0.015 (2)	0.0022 (16)	0.0003 (16)	−0.0033 (18)
C2	0.012 (2)	0.023 (2)	0.020 (2)	0.0018 (18)	0.0005 (18)	0.0047 (19)
C3	0.013 (2)	0.016 (2)	0.027 (3)	−0.0022 (18)	0.0048 (19)	−0.0059 (19)
C4	0.013 (2)	0.016 (2)	0.020 (2)	−0.0016 (18)	−0.0021 (17)	−0.0070 (19)
C5	0.0103 (19)	0.020 (2)	0.017 (2)	0.0040 (17)	0.0032 (17)	−0.0005 (18)
C6	0.0065 (18)	0.017 (2)	0.015 (2)	0.0020 (16)	0.0026 (16)	0.0008 (17)
C7	0.0084 (18)	0.0141 (19)	0.012 (2)	0.0010 (16)	0.0011 (16)	−0.0027 (17)
C8	0.0112 (19)	0.018 (2)	0.014 (2)	0.0017 (17)	0.0039 (17)	0.0002 (18)
C9	0.0075 (19)	0.020 (2)	0.016 (2)	0.0015 (17)	0.0026 (17)	−0.0018 (18)
C10	0.013 (2)	0.023 (2)	0.014 (2)	−0.0004 (18)	0.0044 (17)	−0.0014 (19)
C11	0.014 (2)	0.020 (2)	0.022 (2)	−0.0016 (18)	0.0037 (18)	0.004 (2)
C12	0.013 (2)	0.0138 (19)	0.024 (2)	−0.0029 (17)	0.0018 (18)	−0.0033 (19)
C13	0.013 (2)	0.017 (2)	0.022 (2)	−0.0021 (18)	0.0002 (18)	−0.0036 (19)
C14	0.019 (2)	0.022 (2)	0.020 (2)	−0.0022 (19)	0.0042 (19)	0.001 (2)
C15	0.030 (3)	0.029 (3)	0.022 (3)	−0.007 (2)	−0.002 (2)	0.000 (2)
O8	0.049 (2)	0.0243 (18)	0.033 (2)	0.0018 (18)	0.0193 (19)	−0.0056 (18)

### Geometric parameters (Å, º)

**Table:**

U1—O1	2.206 (3)	C2—H2A	0.9300
U1—O2	1.776 (3)	C2—C3	1.376 (6)
U1—O3	1.777 (3)	C3—H3	0.9300
U1—O4	2.390 (3)	C3—C4	1.386 (6)
U1—O5	2.467 (3)	C4—H4	0.9300
U1—N1	2.489 (4)	C4—C5	1.378 (6)
U1—N4	2.658 (4)	C5—H5	0.9300
O1—C1	1.319 (5)	C5—C6	1.394 (6)
O4—H4A	0.860 (2)	C6—C7	1.461 (5)
O4—H4B	0.860 (2)	C8—C9	1.451 (6)
O5—N5	1.313 (4)	C9—C10	1.385 (6)
O6—N5	1.226 (5)	C10—H10	0.9300
O7—N5	1.221 (5)	C10—C11	1.383 (6)
N1—C7	1.336 (5)	C11—H11	0.9300
N1—C8	1.360 (5)	C11—C12	1.368 (6)
N2—H2	0.8600	C12—H12	0.9300
N2—N3	1.357 (5)	C12—C13	1.391 (6)
N2—C7	1.336 (5)	C13—H13	0.9300
N3—C8	1.314 (5)	C14—C15	1.453 (6)
N4—C9	1.360 (5)	C15—H15A	0.9600
N4—C13	1.347 (5)	C15—H15B	0.9600
N6—C14	1.146 (6)	C15—H15C	0.9600
C1—C2	1.413 (6)	O8—H8A	0.860 (2)
C1—C6	1.405 (6)	O8—H8B	0.860 (2)
			
O1—U1—O4	152.83 (11)	C3—C2—C1	120.4 (4)
O1—U1—O5	77.17 (10)	C3—C2—H2A	119.8
O1—U1—N1	68.61 (11)	C2—C3—H3	119.3
O1—U1—N4	132.17 (11)	C2—C3—C4	121.5 (4)
O2—U1—O1	90.45 (12)	C4—C3—H3	119.3
O2—U1—O3	175.36 (14)	C3—C4—H4	120.6
O2—U1—O4	89.24 (12)	C5—C4—C3	118.8 (4)
O2—U1—O5	100.84 (12)	C5—C4—H4	120.6
O2—U1—N1	91.78 (13)	C4—C5—H5	119.3
O2—U1—N4	90.02 (12)	C4—C5—C6	121.3 (4)
O3—U1—O1	94.15 (13)	C6—C5—H5	119.3
O3—U1—O4	86.96 (12)	C1—C6—C7	118.7 (4)
O3—U1—O5	80.83 (12)	C5—C6—C1	120.1 (4)
O3—U1—N1	89.32 (13)	C5—C6—C7	121.2 (4)
O3—U1—N4	86.43 (13)	N1—C7—C6	126.9 (4)
O4—U1—O5	76.21 (10)	N2—C7—N1	107.7 (4)
O4—U1—N1	138.56 (11)	N2—C7—C6	125.3 (4)
O4—U1—N4	75.00 (11)	N1—C8—C9	120.5 (4)
O5—U1—N1	143.57 (10)	N3—C8—N1	113.7 (4)
O5—U1—N4	149.01 (10)	N3—C8—C9	125.7 (4)
N1—U1—N4	63.57 (10)	N4—C9—C8	114.9 (4)
C1—O1—U1	149.5 (3)	N4—C9—C10	122.4 (4)
U1—O4—H4A	129 (4)	C10—C9—C8	122.7 (4)
U1—O4—H4B	126 (3)	C9—C10—H10	120.6
H4A—O4—H4B	104 (4)	C11—C10—C9	118.8 (4)
N5—O5—U1	124.2 (2)	C11—C10—H10	120.6
C7—N1—U1	133.5 (3)	C10—C11—H11	120.2
C7—N1—C8	104.5 (4)	C12—C11—C10	119.6 (4)
C8—N1—U1	122.0 (3)	C12—C11—H11	120.2
N3—N2—H2	124.3	C11—C12—H12	120.5
C7—N2—H2	124.3	C11—C12—C13	119.0 (4)
C7—N2—N3	111.5 (3)	C13—C12—H12	120.5
C8—N3—N2	102.6 (3)	N4—C13—C12	122.7 (4)
C9—N4—U1	118.9 (3)	N4—C13—H13	118.6
C13—N4—U1	123.6 (3)	C12—C13—H13	118.6
C13—N4—C9	117.5 (4)	N6—C14—C15	178.4 (5)
O6—N5—O5	116.9 (4)	C14—C15—H15A	109.5
O7—N5—O5	118.6 (4)	C14—C15—H15B	109.5
O7—N5—O6	124.6 (4)	C14—C15—H15C	109.5
O1—C1—C2	119.5 (4)	H15A—C15—H15B	109.5
O1—C1—C6	122.5 (4)	H15A—C15—H15C	109.5
C6—C1—C2	118.0 (4)	H15B—C15—H15C	109.5
C1—C2—H2A	119.8	H8A—O8—H8B	102 (6)
			
U1—O1—C1—C2	172.3 (4)	C1—C6—C7—N1	4.2 (6)
U1—O1—C1—C6	−7.1 (8)	C1—C6—C7—N2	−177.7 (4)
U1—O5—N5—O6	175.9 (2)	C2—C1—C6—C5	−0.3 (6)
U1—O5—N5—O7	−4.3 (5)	C2—C1—C6—C7	180.0 (4)
U1—N1—C7—N2	179.1 (3)	C2—C3—C4—C5	−0.1 (6)
U1—N1—C7—C6	−2.6 (6)	C3—C4—C5—C6	−0.8 (6)
U1—N1—C8—N3	−179.2 (3)	C4—C5—C6—C1	1.0 (6)
U1—N1—C8—C9	1.8 (5)	C4—C5—C6—C7	−179.3 (4)
U1—N4—C9—C8	−0.6 (4)	C5—C6—C7—N1	−175.5 (4)
U1—N4—C9—C10	179.5 (3)	C5—C6—C7—N2	2.6 (6)
U1—N4—C13—C12	−179.3 (3)	C6—C1—C2—C3	−0.6 (6)
O1—C1—C2—C3	−180.0 (4)	C7—N1—C8—N3	−0.1 (5)
O1—C1—C6—C5	179.0 (4)	C7—N1—C8—C9	−179.2 (4)
O1—C1—C6—C7	−0.7 (6)	C7—N2—N3—C8	0.1 (4)
N1—C8—C9—N4	−0.7 (5)	C8—N1—C7—N2	0.2 (4)
N1—C8—C9—C10	179.2 (4)	C8—N1—C7—C6	178.5 (4)
N2—N3—C8—N1	0.0 (4)	C8—C9—C10—C11	−179.8 (4)
N2—N3—C8—C9	179.0 (4)	C9—N4—C13—C12	0.6 (6)
N3—N2—C7—N1	−0.2 (5)	C9—C10—C11—C12	0.1 (6)
N3—N2—C7—C6	−178.6 (4)	C10—C11—C12—C13	0.0 (6)
N3—C8—C9—N4	−179.6 (4)	C11—C12—C13—N4	−0.4 (6)
N3—C8—C9—C10	0.2 (6)	C13—N4—C9—C8	179.4 (4)
N4—C9—C10—C11	0.1 (6)	C13—N4—C9—C10	−0.4 (6)
C1—C2—C3—C4	0.8 (6)		

### Hydrogen-bond geometry (Å, º)

**Table:**

*D*—H···*A*	*D*—H	H···*A*	*D*···*A*	*D*—H···*A*
O4—H4*A*···O5^i^	0.86 (1)	1.92 (2)	2.752 (4)	162 (5)
O4—H4*B*···O8	0.86 (1)	1.73 (1)	2.581 (5)	168 (5)
N2—H2···N6	0.86	2.07	2.909 (5)	165
O8—H8*A*···N3^ii^	0.86 (1)	2.05 (2)	2.890 (5)	164 (6)
O8—H8*B*···O6^iii^	0.86 (1)	2.22 (3)	3.001 (5)	150 (6)

Symmetry codes: (i) −*x*+2, −*y*+1, −*z*+1; (ii) *x*, −*y*+3/2, *z*+1/2; (iii) *x*, *y*+1, *z*.
